# The genome sequence of the Long-tailed duck, *Clangula hyemalis *(Linnaeus, 1758)

**DOI:** 10.12688/wellcomeopenres.22779.2

**Published:** 2024-10-15

**Authors:** Michelle F. O’Brien, Rosa Lopez Colom, Natural History Museum Genome Acquisition Lab, Darwin Tree of Life Barcoding collective, Wellcome Sanger Institute Tree of Life Management, Samples and Laboratory team, Wellcome Sanger Institute Scientific Operations: Sequencing Operations, Wellcome Sanger Institute Tree of Life Core Informatics team, Tree of Life Core Informatics collective, Darwin Tree of Life Consortium

**Affiliations:** 1 Wildfowl & Wetlands Trust, Slimbridge, England, UK

**Keywords:** Clangula hyemalis, Long-tailed duck, genome sequence, chromosomal, Anseriformes

## Abstract

We present a genome assembly from an individual male *Clangula hyemalis* (the Long-tailed duck; Chordata; Aves; Anseriformes; Anatidae). The genome sequence spans 1,206.10 megabases. Most of the assembly is scaffolded into 41 chromosomal pseudomolecules, including the Z sex chromosome. The mitochondrial genome has also been assembled and is 16.63 kilobases in length.

## Species taxonomy

Eukaryota; Opisthokonta; Metazoa; Eumetazoa; Bilateria; Deuterostomia; Chordata; Craniata; Vertebrata; Gnathostomata; Teleostomi; Euteleostomi; Sarcopterygii; Dipnotetrapodomorpha; Tetrapoda; Amniota; Sauropsida; Sauria; Archelosauria; Archosauria; Dinosauria; Saurischia; Theropoda; Coelurosauria; Aves; Neognathae; Galloanserae; Anseriformes; Anatidae; Anatinae; *Clangula*; *Clangula hyemalis* (Linnaeus, 1758) (NCBI:txid197941).

## Background

Long-tailed ducks ( *Clangula hyemalis*) have rounded heads, small bills and short necks. Their name derives from their long, pointed tail which is seen in males in both summer and winter. These elongated feathers can add up to 13 cm to their body length of 40–47 cm ( Gooders & Boyer, 1986). Males can reach a weight of 1100 g and females 950 g ( Robertson & Savard, 2020).

*C*. *hyemalis* have an extremely complex plumage pattern with different male and female plumage but also distinct summer and winter plumage and different plumage when in eclipse (female-like plumage worn by the male for approximately a month in summer post-breeding) and during their first year. Plumage is mostly mottled brown and buff dorsally and white ventrally although in winter males are black and white dorsally. Males have a dark brown breast which, during summer and eclipse, extends over the head leaving a pale area around the eye ( Gooders & Boyer, 1986).

Long-tailed ducks are more marine than some other seaducks and, outside the breeding season, spend most of their time out of sight of land. This correlates with their ability to find food at greater depths than other ducks – up to 35 m. Their diet consists of mostly molluscs, crustaceans, insect larvae and fish ( Gooders & Boyer, 1986; Owen, 1977). They nest in shallow scrapes lined with grass and down and the timing of egg laying (of 5 to 7 eggs) varies between locations. Incubation takes 24 to 29 days ( Robertson & Savard, 2020).

The global distribution of *C. hyemalis* is circumpolar – breeding across Alaska, Canada, Greenland, Iceland and across Scandinavia, Northern Siberia to the Bering Straits ( Gooders & Boyer, 1986). They breed on average further north than any other duck ( Owen, 1977). They are short- to medium-distance complete migrants although they may also have resident populations in areas such as Alaska ( Robertson & Savard, 2020). 

This species is at high risk of gillnet bycatch (at least 25,000 from over four decades) in the North and Baltic Seas, which has contributed to significant population declines in these regions ( Bellebaum *et al.*, 2013; Žydelis *et al.*, 2009; Žydelis *et al.*, 2013). LED lights (used as underwater visual deterrents) have been shown to be ineffective when deployed on gillnets, while white flashing lights may even make foraging sites more attractive to Long-tailed ducks ( Cantlay *et al.*, 2020). 

Preliminary studies have also show Long-tailed ducks to be at risk from ingestion of marine debris, including plastics – in one study 5% of individuals in the Baltic Sea caught as bycatch had marine debris present in their gastrointestinal tracts. It has been postulated that this risk may be increased due to this species having a broad isotopic niche ( Morkūnas *et al.*, 2021).

The Long-tailed duck is listed on the IUCN Red List as “vulnerable” globally ( BirdLife International, 2018), but as “least concern” in Europe ( BirdLife International, 2021), despite its population declining.

Here we present a chromosomally complete genome sequence for *Clangula hyemalis*, based on one male specimen from the WWT Arundel Wetland Centre, West Sussex, England, UK.

## Genome sequence report

**Figure 1.**
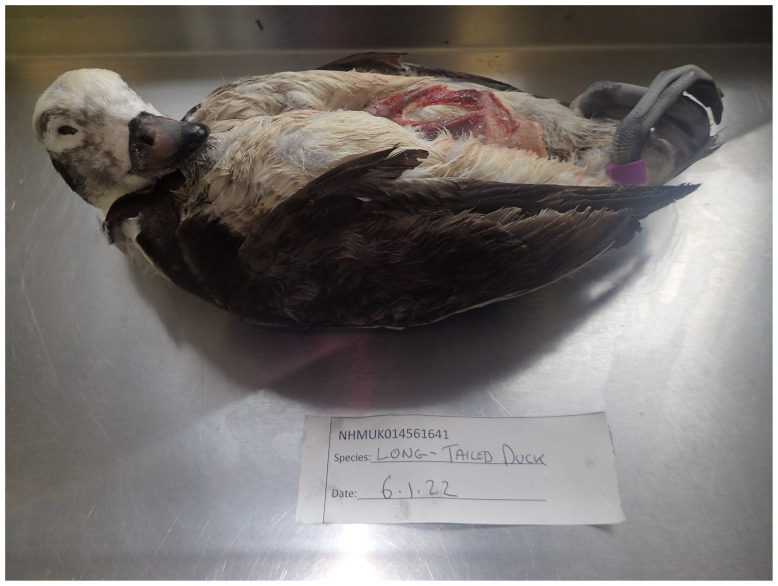
Photograph of the *Clangula hyemalis* (bClaHye2) specimen used for genome sequencing.

The genome of an adult male *Clangula hyemalis* ( Figure 1) was sequenced using Pacific Biosciences single-molecule HiFi long reads, generating a total of 51.40 Gb (gigabases) from 5.13 million reads, providing an estimated 43-fold coverage based on the GenomeScope estimate of the genome size. Primary assembly contigs were scaffolded with chromosome conformation Hi-C data, which produced 53.52 Gb from 354.41 million reads, yielding an approximate coverage of 44-fold. Specimen and sequencing information is summarised in Table 1.

**Table 1.** Specimen and sequencing data for *Clangula hyemalis*.

Project information			
**Study title**	*Clangula hyemalis* (long-tailed duck)		
**Umbrella BioProject**	PRJEB68290		
**Species**	*Clangula hyemalis*		
**BioSample**	SAMEA112468035		
**NCBI taxonomy ID**	197941		
Specimen information			
**Technology**	**ToLID**	**BioSample accession**	**Organism part**
**PacBio long read sequencing**	bClaHye2	SAMEA112468076	muscle
**Hi-C sequencing**	bClaHye2	SAMEA112468076	muscle
**RNA sequencing**	bClaHye2	SAMEA112468076	muscle
Sequencing information			
**Platform**	**Run accession**	**Read count**	**Base count (Gb)**
**Hi-C Illumina NovaSeq 6000**	ERR12259843	3.54e+08	53.52
**PacBio Sequel IIe**	ERR12257420	2.38e+06	22.73
**PacBio Sequel IIe**	ERR12257421	2.75e+06	28.67
**RNA Illumina NovaSeq 6000**	ERR12321243	6.77e+07	10.22

**Figure 2.**
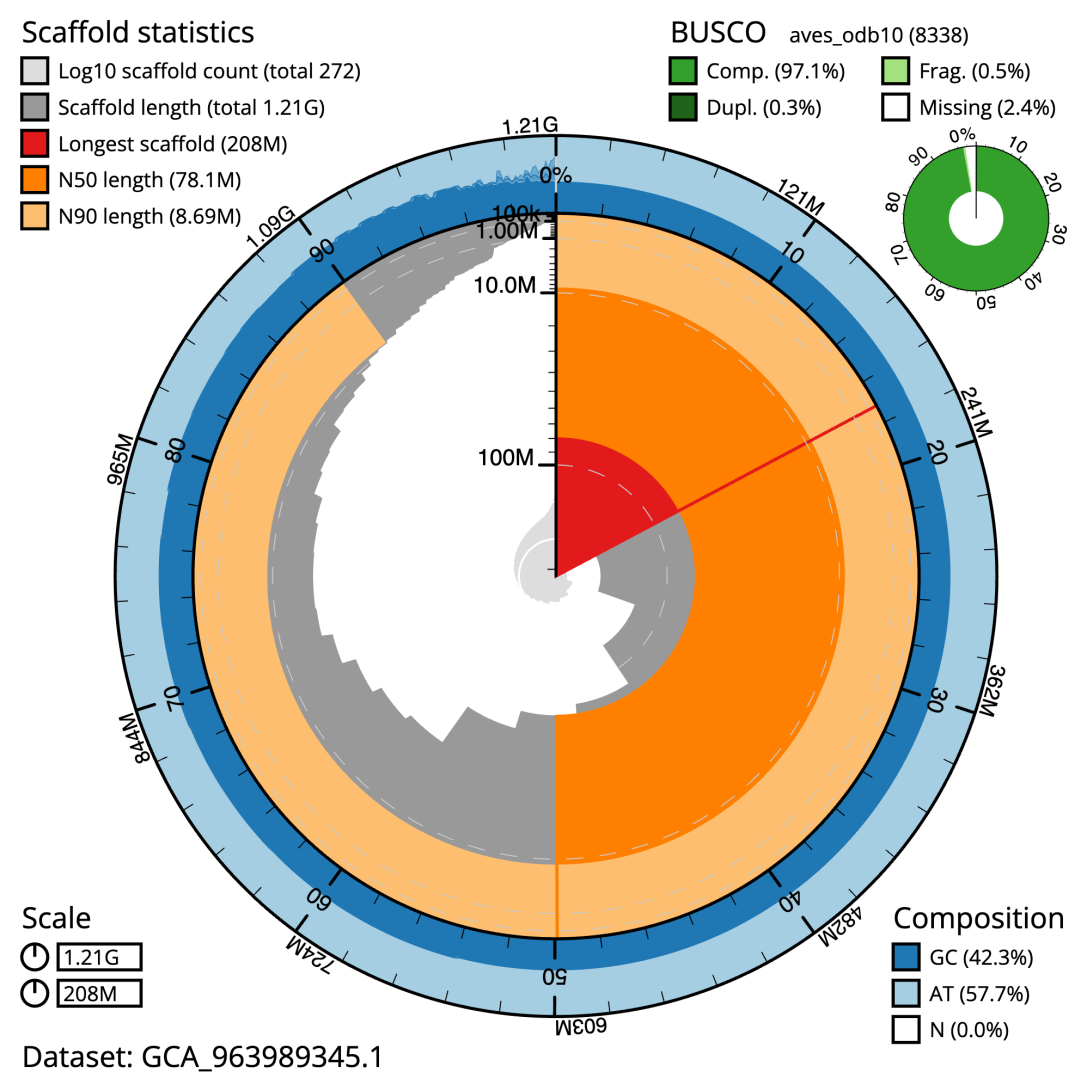
Genome assembly of *Clangula hyemalis*, bClaHye2.1: metrics. The BlobToolKit snail plot shows N50 metrics and BUSCO gene completeness. The main plot is divided into 1,000 size-ordered bins around the circumference with each bin representing 0.1% of the 1,206,104,048 bp assembly. The distribution of scaffold lengths is shown in dark grey with the plot radius scaled to the longest scaffold present in the assembly (207,631,701 bp, shown in red). Orange and pale-orange arcs show the N50 and N90 scaffold lengths (78,069,974 and 8,686,370 bp), respectively. The pale grey spiral shows the cumulative scaffold count on a log scale with white scale lines showing successive orders of magnitude. The blue and pale-blue area around the outside of the plot shows the distribution of GC, AT and N percentages in the same bins as the inner plot. A summary of complete, fragmented, duplicated and missing BUSCO genes in the aves_odb10 set is shown in the top right. An interactive version of this figure is available at https://blobtoolkit.genomehubs.org/view/Clangula_hyemalis/dataset/GCA_963989345.1/snail.

**Figure 3.**
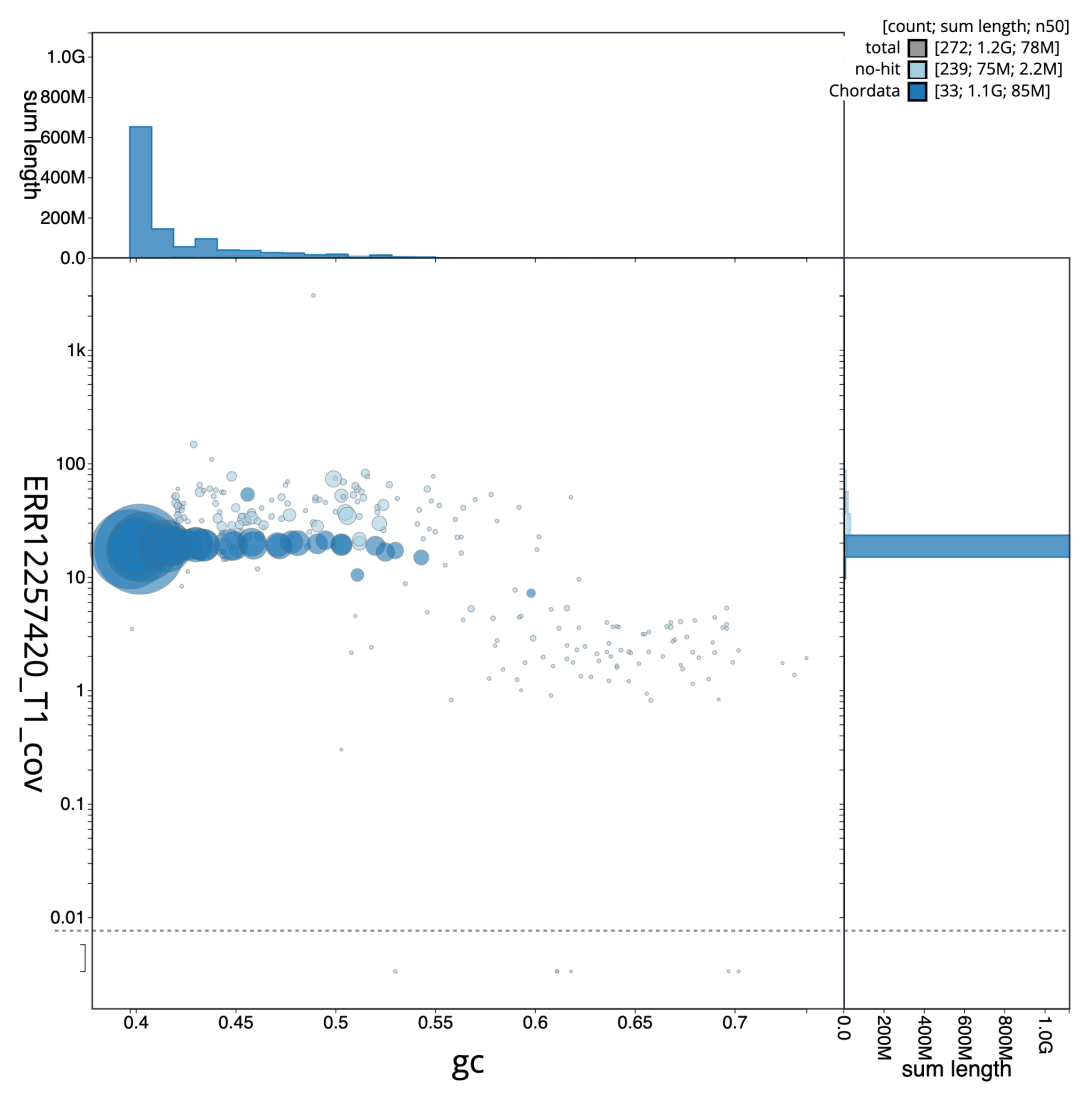
Genome assembly of *Clangula hyemalis*, bClaHye2.1: BlobToolKit GC-coverage plot. Sequences are coloured by phylum. Circles are sized in proportion to sequence length. Histograms show the distribution of sequence length sum along each axis. An interactive version of this figure is available at https://blobtoolkit.genomehubs.org/view/Clangula_hyemalis/dataset/GCA_963989345.1/blob.

**Figure 4.**
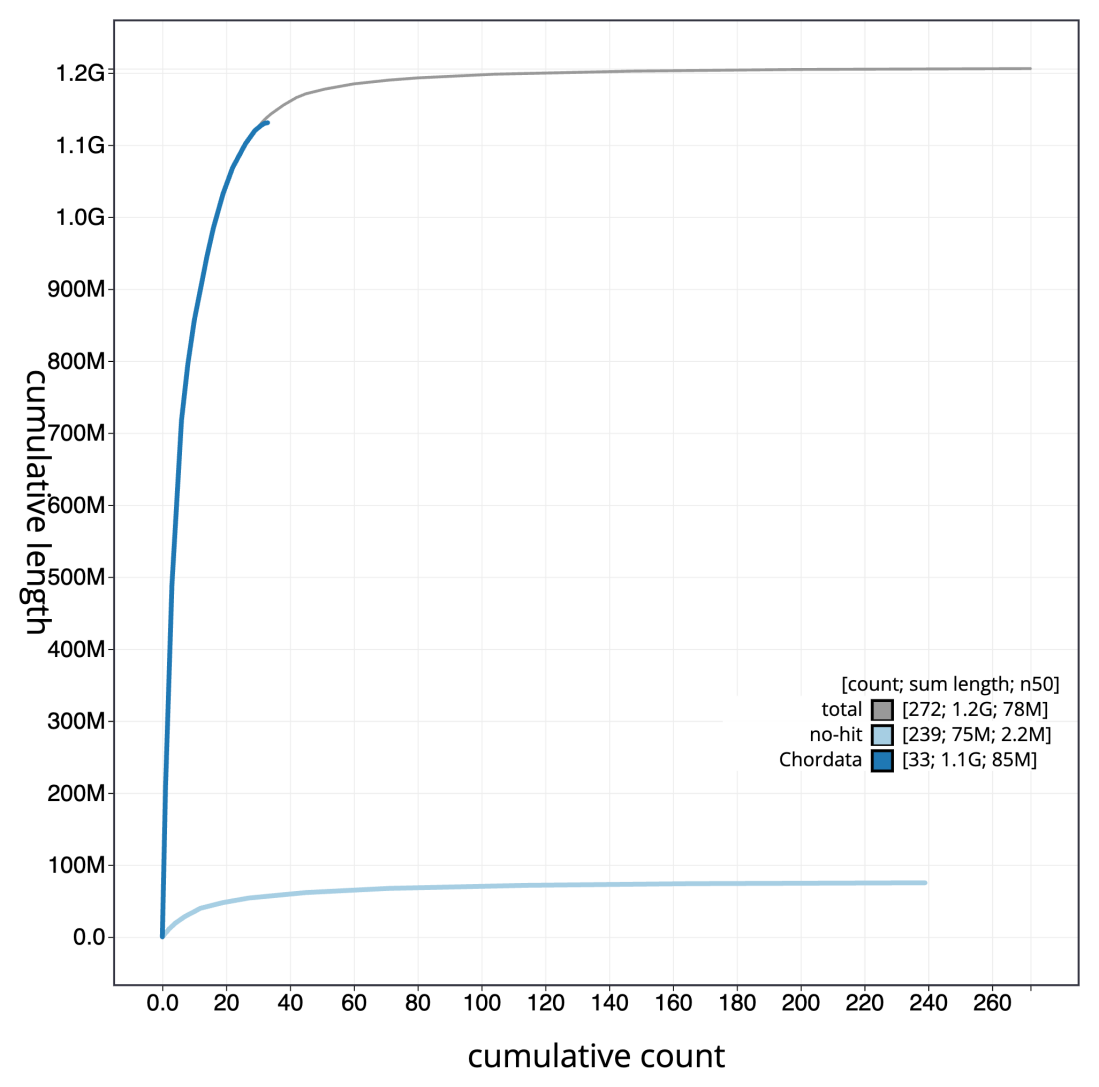
Genome assembly of *Clangula hyemalis* bClaHye2.1: BlobToolKit cumulative sequence plot. The grey line shows cumulative length for all sequences. Coloured lines show cumulative lengths of sequences assigned to each phylum using the buscogenes taxrule. An interactive version of this figure is available at https://blobtoolkit.genomehubs.org/view/Clangula_hyemalis/dataset/GCA_963989345.1/cumulative.

**Figure 5.**
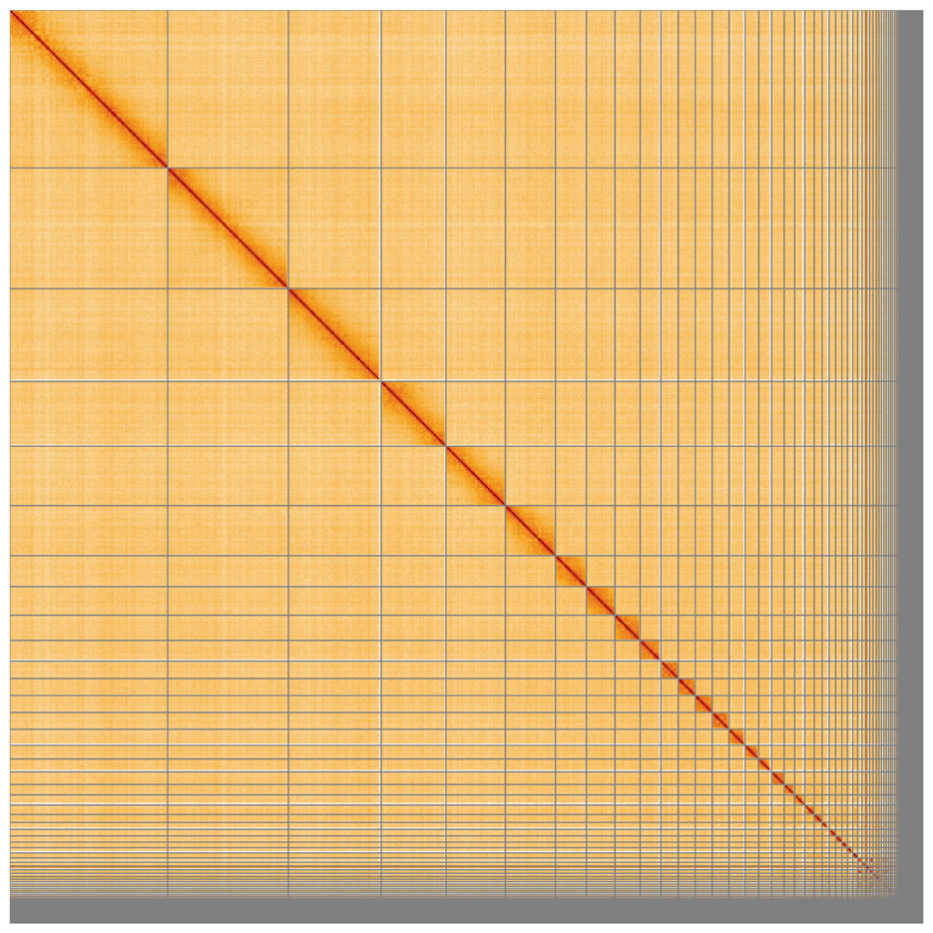
Genome assembly of *Clangula hyemalis* bClaHye2.1: Hi-C contact map of the bClaHye2.1 assembly, visualised using HiGlass. Chromosomes are shown in order of size from left to right and top to bottom. An interactive version of this figure may be viewed at https://genome-note-higlass.tol.sanger.ac.uk/l/?d=D62vjOvyTCauGdqPLb4xXg.

Manual assembly curation corrected 50 missing joins or mis-joins and two haplotypic duplications, reducing the scaffold number by 12.54%. The final assembly has a total length of 1,206.10 Mb in 271 sequence scaffolds with a scaffold N50 of 78.1 Mb ( Table 2), with 544 gaps. The snail plot in Figure 2 provides a summary of the assembly statistics, while the distribution of assembly scaffolds on GC proportion and coverage is shown in Figure 3. The cumulative assembly plot in Figure 4 shows curves for subsets of scaffolds assigned to different phyla. Most (95.1%) of the assembly sequence was assigned to 41 chromosomal-level scaffolds, representing 40 autosomes and the Z sex chromosome. Chromosome-scale scaffolds confirmed by the Hi-C data are named in order of size ( Figure 5; Table 3). While not fully phased, the assembly deposited is of one haplotype. Contigs corresponding to the second haplotype have also been deposited. The mitochondrial genome was also assembled and can be found as a contig within the multifasta file of the genome submission.

**Table 2.** Genome assembly data for *Clangula hyemalis*, bClaHye2.1.

Genome assembly		
Assembly name	bClaHye2.1	
Assembly accession	GCA_963989345.1	
*Accession of alternate haplotype*	*GCA_963989315.1*	
Span (Mb)	1,206.10	
Number of contigs	816	
Contig N50 length (Mb)	3.7	
Number of scaffolds	271	
Scaffold N50 length (Mb)	78.1	
Longest scaffold (Mb)	207.7	
Assembly metrics *		*Benchmark*
Consensus quality (QV)	60.1	*≥ 50*
*k*-mer completeness	100.0%	*≥ 95%*
BUSCO **	C:97.1%[S:96.9%,D:0.2%], F:0.5%,M:2.4%,n:8,338	*C ≥ 95%*
Percentage of assembly mapped to chromosomes	95.1%	*≥ 95%*
Sex chromosomes	Z	*localised homologous pairs*
Organelles	Mitochondrial genome: 16.63 kb	*complete single alleles*

* Assembly metric benchmarks are adapted from column VGP-2020 of “Table 1: Proposed standards and metrics for defining genome assembly quality” from Rhie *et al.* (2021). ** BUSCO scores based on the vertebrata_odb10 BUSCO set using version 5.4.3. C = complete [S = single copy, D = duplicated], F = fragmented, M = missing, n = number of orthologues in comparison. A full set of BUSCO scores is available at https://blobtoolkit.genomehubs.org/view/Clangula_hyemalis/dataset/GCA_963989345.1/busco.

**Table 3.** Chromosomal pseudomolecules in the genome assembly of *Clangula hyemalis*, bClaHye2.

INSDC accession	Name	Length (Mb)	GC%
OZ022426.1	1	207.63	40.0
OZ022427.1	2	158.76	39.5
OZ022428.1	3	121.98	40.5
OZ022430.1	4	78.07	40.0
OZ022431.1	5	65.83	41.5
OZ022432.1	6	40.67	41.5
OZ022433.1	7	37.69	41.5
OZ022434.1	8	33.21	42.0
OZ022435.1	9	27.11	43.0
OZ022436.1	10	22.8	43.0
OZ022437.1	11	22.37	43.5
OZ022438.1	12	22.36	43.5
OZ022439.1	13	22.09	42.5
OZ022440.1	14	21.11	44.5
OZ022441.1	15	17.94	45.0
OZ022442.1	16	17.02	46.0
OZ022443.1	17	16.52	46.0
OZ022444.1	18	13.64	47.0
OZ022445.1	19	13.34	48.0
OZ022446.1	20	12.44	47.0
OZ022447.1	21	10.46	48.0
OZ022448.1	22	9.08	50.5
OZ022449.1	23	8.69	50.5
OZ022450.1	24	8.13	49.0
OZ022451.1	25	7.27	52.0
OZ022452.1	26	7.0	49.5
OZ022453.1	27	6.53	52.5
OZ022454.1	28	5.08	53.0
OZ022455.1	29	4.05	54.5
OZ022456.1	30	3.73	52.0
OZ022457.1	31	3.27	45.5
OZ022458.1	32	3.24	51.0
OZ022459.1	33	3.11	51.0
OZ022460.1	34	2.7	51.0
OZ022461.1	35	2.55	47.5
OZ022462.1	36	2.23	49.0
OZ022463.1	37	0.89	60.0
OZ022464.1	38	0.34	57.0
OZ022465.1	39	0.25	60.0
OZ022466.1	40	0.22	61.5
OZ022429.1	Z	85.19	40.0
OZ022467.1	MT	0.02	49.0

The estimated Quality Value (QV) of the final assembly is 60.1 with *k*-mer completeness of 100.0%, and the assembly has a BUSCO v5.4.3 completeness of 97.1% (single = 96.9%, duplicated = 0.2%), using the vertebrata_odb10 reference set ( *n* = 8,338).

Metadata for specimens, BOLD barcode results, spectra estimates, sequencing runs, contaminants and pre-curation assembly statistics are given at https://links.tol.sanger.ac.uk/species/197941.

## Methods

### Sample acquisition and nucleic acid extraction

An adult male *Clangula hyemalis* (specimen ID NHMUK014561641, ToLID bClaHye2), a captive specimen, was found deceased at WWT Arundel, West Sussex (latitude 50.86, longitude –0.55) on 2022-01-06. The specimen was collected and identified by Michelle O'Brien (Wildfowl & Wetlands Trust). Several small samples of pectoral muscle were taken from a captive specimen in 2022 and stored at –20°C.

The workflow for high molecular weight (HMW) DNA extraction at the Wellcome Sanger Institute (WSI) Tree of Life Core Laboratory includes a sequence of core procedures: sample preparation; sample homogenisation, DNA extraction, fragmentation, and clean-up. In sample preparation, the bClaHye2 sample was weighed and dissected on dry ice ( Jay *et al.*, 2023). For sample homogenisation, muscle tissue was cryogenically disrupted using the Covaris cryoPREP ^®^ Automated Dry Pulverizer ( Narváez-Gómez *et al.*, 2023).

HMW DNA was extracted at the WSI Scientific Operations core using the Automated MagAttract v2 protocol ( Oatley *et al.*, 2023). The DNA was sheared into an average fragment size of 12–20 kb in a Megaruptor 3 system with speed setting 31 ( Bates *et al.*, 2023). Sheared DNA was purified by solid-phase reversible immobilisation ( Strickland *et al.*, 2023): in brief, the method employs of AMPure PB beads to eliminate shorter fragments and concentrate the DNA. The concentration of the sheared and purified DNA was assessed using a Nanodrop spectrophotometer and Qubit Fluorometer using the Qubit dsDNA High Sensitivity Assay kit. Fragment size distribution was evaluated by running the sample on the FemtoPulse system.

RNA was extracted from muscle tissue of bClaHye2 in the Tree of Life Laboratory at the WSI using the RNA Extraction: Automated MagMax™ *mir*Vana protocol ( do Amaral *et al.*, 2023). The RNA concentration was assessed using a Nanodrop spectrophotometer and a Qubit Fluorometer using the Qubit RNA Broad-Range Assay kit. Analysis of the integrity of the RNA was done using the Agilent RNA 6000 Pico Kit and Eukaryotic Total RNA assay.

Protocols developed by the WSI Tree of Life laboratory are publicly available on protocols.io ( Denton *et al.*, 2023).

### Hi-C preparation

Tissue from the muscle of the bClaHye2sample was processed at the WSI Scientific Operations core, using the Arima-HiC v2 kit. In brief, frozen tissue (stored at –80°C) was fixed, and the DNA crosslinked using a TC buffer with 22% formaldehyde. After crosslinking, the tissue was homogenised using the Diagnocine Power Masher-II and BioMasher-II tubes and pestles. Following the kit manufacturer's instructions, crosslinked DNA was digested using a restriction enzyme master mix. The 5’-overhangs were then filled in and labelled with biotinylated nucleotides and proximally ligated. An overnight incubation was carried out for enzymes to digest remaining proteins and for crosslinks to reverse. A clean up was performed with SPRIselect beads prior to library preparation.

### Library preparation and sequencing

Library preparation and sequencing were performed at the WSI Scientific Operations core. Pacific Biosciences HiFi circular consensus DNA sequencing libraries were prepared using the PacBio Express Template Preparation Kit v2.0 (Pacific Biosciences, California, USA) as per the manufacturer's instructions. The kit includes the reagents required for removal of single-strand overhangs, DNA damage repair, end repair/A-tailing, adapter ligation, and nuclease treatment. Library preparation also included a library purification step using AMPure PB beads (Pacific Biosciences, California, USA) and size selection step to remove templates shorter than 3 kb using AMPure PB modified SPRI. DNA concentration was quantified using the Qubit Fluorometer v2.0 and Qubit HS Assay Kit and the final library fragment size analysis was carried out using the Agilent Femto Pulse Automated Pulsed Field CE Instrument and gDNA 165kb gDNA and 55kb BAC analysis kit. Samples were sequenced using the Sequel IIe system (Pacific Biosciences, California, USA). The concentration of the library loaded onto the Sequel IIe was between 40–135 pM. The SMRT link software, a PacBio web-based end-to-end workflow manager, was used to set-up and monitor the run, as well as perform primary and secondary analysis of the data upon completion.

Poly(A) RNA-Seq libraries were constructed using the NEB Ultra II RNA Library Prep kit, following the manufacturer’s instructions. RNA sequencing was performed on the Illumina NovaSeq 6000 instrument.

For Hi-C library preparation, DNA was fragmented to a size of 400 to 600 bp using a Covaris E220 sonicator. The DNA was then enriched, barcoded, and amplified using the NEBNext Ultra II DNA Library Prep Kit following manufacturers’ instructions. The Hi-C sequencing was performed using paired-end sequencing with a read length of 150 bp on an Illumina NovaSeq 6000 instrument.

### Genome assembly, curation and evaluation

***Assembly***

The original assembly of HiFi reads was performed using Hifiasm ( Cheng *et al.*, 2021) with the --primary option. Haplotypic duplications were identified and removed with purge_dups ( Guan *et al.*, 2020). Hi-C reads are further mapped with bwa-mem2 ( Vasimuddin *et al.*, 2019) to the primary contigs, which are further scaffolded using the provided Hi-C data ( Rao *et al.*, 2014) in YaHS ( Zhou *et al.*, 2023) using the --break option. Scaffolded assemblies are evaluated using Gfastats ( Formenti *et al.*, 2022), BUSCO ( Manni *et al.*, 2021) and MERQURY.FK ( Rhie *et al.*, 2020).

The mitochondrial genome was assembled using MitoHiFi ( Uliano-Silva *et al.*, 2023), which runs MitoFinder ( Allio *et al.*, 2020) and uses these annotations to select the final mitochondrial contig and to ensure the general quality of the sequence.

***Assembly curation***

The assembly was decontaminated using the Assembly Screen for Cobionts and Contaminants (ASCC) pipeline (article in preparation). Flat files and maps used in curation were generated in TreeVal ( Pointon *et al.*, 2023). Manual curation was primarily conducted using PretextView ( Harry, 2022), with additional insights provided by JBrowse2 ( Diesh *et al.*, 2023) and HiGlass ( Kerpedjiev *et al.*, 2018). Scaffolds were visually inspected and corrected as described by Howe *et al*. (2021). Any identified contamination, missed joins, and mis-joins were corrected, and duplicate sequences were tagged and removed. The entire process is documented at https://gitlab.com/wtsi-grit/rapid-curation (article in preparation).

### Evaluation of the final assembly

The final assembly was post-processed and evaluated with the three Nextflow ( Di Tommaso *et al.*, 2017) DSL2 pipelines “sanger-tol/readmapping” ( Surana *et al.*, 2023a), “sanger-tol/genomenote” ( Surana *et al.*, 2023b), and “sanger-tol/blobtoolkit” ( Muffato *et al.*, 2024). The pipeline sanger-tol/readmapping aligns the Hi-C reads with bwa-mem2 ( Vasimuddin *et al.*, 2019) and combines the alignment files with SAMtools ( Danecek *et al.*, 2021). The sanger-tol/genomenote pipeline transforms the Hi-C alignments into a contact map with BEDTools ( Quinlan & Hall, 2010) and the Cooler tool suite ( Abdennur & Mirny, 2020), which is then visualised with HiGlass ( Kerpedjiev *et al.*, 2018). It also provides statistics about the assembly with the NCBI datasets ( Sayers *et al.*, 2024) report, computes *k*-mer completeness and QV consensus quality values with FastK and MERQURY.FK, and a completeness assessment with BUSCO ( Manni *et al.*, 2021).

The sanger-tol/blobtoolkit pipeline is a Nextflow port of the previous Snakemake Blobtoolkit pipeline ( Challis *et al.*, 2020). It aligns the PacBio reads with SAMtools and minimap2 ( Li, 2018) and generates coverage tracks for regions of fixed size. In parallel, it queries the GoaT database ( Challis *et al.*, 2023) to identify all matching BUSCO lineages to run BUSCO ( Manni *et al.*, 2021). For the three domain-level BUSCO lineage, the pipeline aligns the BUSCO genes to the Uniprot Reference Proteomes database ( Bateman *et al.*, 2023) with DIAMOND ( Buchfink *et al.*, 2021) blastp. The genome is also split into chunks according to the density of the BUSCO genes from the closest taxonomically lineage, and each chunk is aligned to the Uniprot Reference Proteomes database with DIAMOND blastx. Genome sequences that have no hit are then chunked with seqtk and aligned to the NT database with blastn ( Altschul *et al.*, 1990). All those outputs are combined with the blobtools suite into a blobdir for visualisation.

The genome assembly and evaluation pipelines were developed using the nf-core tooling ( Ewels *et al.*, 2020), use MultiQC ( Ewels *et al.*, 2016), and make extensive use of the Conda package manager, the Bioconda initiative ( Grüning *et al.*, 2018), the Biocontainers infrastructure ( da Veiga Leprevost *et al.*, 2017), and the Docker ( Merkel, 2014) and Singularity ( Kurtzer *et al.*, 2017) containerisation solutions.

Table 4 contains a list of relevant software tool versions and sources.

**Table 4.** Software tools: versions and sources.

Software tool	Version	Source
BEDTools	2.30.0	https://github.com/arq5x/bedtools2
BLAST	2.14.0	ftp://ftp.ncbi.nlm.nih.gov/blast/executables/blast+/
BlobToolKit	4.3.7	https://github.com/blobtoolkit/blobtoolkit
BUSCO	5.4.3 and 5.5.0	https://gitlab.com/ezlab/busco
bwa-mem2	2.2.1	https://github.com/bwa-mem2/bwa-mem2
Cooler	0.8.11	https://github.com/open2c/cooler
DIAMOND	2.1.8	https://github.com/bbuchfink/diamond
fasta_windows	0.2.4	https://github.com/tolkit/fasta_windows
FastK	427104ea91c78c3b8b8b49f1a7d6bbeaa869ba1c	https://github.com/thegenemyers/FASTK
Gfastats	1.3.6	https://github.com/vgl-hub/gfastats
GoaT CLI	0.2.5	https://github.com/genomehubs/goat-cli
Hifiasm	0.19.5-r587	https://github.com/chhylp123/hifiasm
HiGlass	44086069ee7d4d3f6f3f0012569789ec138f42b84 aa44357826c0b6753eb28de	https://github.com/higlass/higlass
Merqury.FK	d00d98157618f4e8d1a9190026b19b471055b22e	https://github.com/thegenemyers/MERQURY.FK
MitoHiFi	3	https://github.com/marcelauliano/MitoHiFi
MultiQC	1.14, 1.17, and 1.18	https://github.com/MultiQC/MultiQC
NCBI Datasets	15.12.0	https://github.com/ncbi/datasets
Nextflow	23.04.0-5857	https://github.com/nextflow-io/nextflow
PretextView	0.2	https://github.com/sanger-tol/PretextView
purge_dups	1.2.5	https://github.com/dfguan/purge_dups
samtools	1.16.1, 1.17, and 1.18	https://github.com/samtools/samtools
sanger-tol/ascc	-	https://github.com/sanger-tol/ascc
sanger-tol/genomenote	1.1.1	https://github.com/sanger-tol/genomenote
sanger-tol/readmapping	1.2.1	https://github.com/sanger-tol/readmapping
Seqtk	1.3	https://github.com/lh3/seqtk
Singularity	3.9.0	https://github.com/sylabs/singularity
TreeVal	1.0.0	https://github.com/sanger-tol/treeval
YaHS	1.2a.2	https://github.com/c-zhou/yahs

### Wellcome Sanger Institute – Legal and Governance

The materials that have contributed to this genome note have been supplied by a Darwin Tree of Life Partner. The submission of materials by a Darwin Tree of Life Partner is subject to the **‘Darwin Tree of Life Project Sampling Code of Practice’**, which can be found in full on the Darwin Tree of Life website here. By agreeing with and signing up to the Sampling Code of Practice, the Darwin Tree of Life Partner agrees they will meet the legal and ethical requirements and standards set out within this document in respect of all samples acquired for, and supplied to, the Darwin Tree of Life Project. 

Further, the Wellcome Sanger Institute employs a process whereby due diligence is carried out proportionate to the nature of the materials themselves, and the circumstances under which they have been/are to be collected and provided for use. The purpose of this is to address and mitigate any potential legal and/or ethical implications of receipt and use of the materials as part of the research project, and to ensure that in doing so we align with best practice wherever possible. The overarching areas of consideration are:

• Ethical review of provenance and sourcing of the material

• Legality of collection, transfer and use (national and international) 

Each transfer of samples is further undertaken according to a Research Collaboration Agreement or Material Transfer Agreement entered into by the Darwin Tree of Life Partner, Genome Research Limited (operating as the Wellcome Sanger Institute), and in some circumstances other Darwin Tree of Life collaborators.

## Data Availability

European Nucleotide Archive: *Clangula hyemalis* (long-tailed duck). Accession number PRJEB68290; https://identifiers.org/ena.embl/PRJEB68290 ( Wellcome Sanger Institute, 2024). The genome sequence is released openly for reuse. The *Clangula hyemalis* genome sequencing initiative is part of the Darwin Tree of Life (DToL) project. All raw sequence data and the assembly have been deposited in INSDC databases. The genome will be annotated using available RNA-Seq data and presented through the Ensembl pipeline at the European Bioinformatics Institute. Raw data and assembly accession identifiers are reported in Table 1 and Table 2.

## Author information

Members of the Natural History Museum Genome Acquisition Lab are listed here: https://doi.org/10.5281/zenodo.12159242.

Members of the Darwin Tree of Life Barcoding collective are listed here: https://doi.org/10.5281/zenodo.12158331

Members of the Wellcome Sanger Institute Tree of Life Management, Samples and Laboratory team are listed here: https://doi.org/10.5281/zenodo.12162482.

Members of Wellcome Sanger Institute Scientific Operations: Sequencing Operations are listed here: https://doi.org/10.5281/zenodo.12165051.

Members of the Wellcome Sanger Institute Tree of Life Core Informatics team are listed here: https://doi.org/10.5281/zenodo.12160324.

Members of the Tree of Life Core Informatics collective are listed here: https://doi.org/10.5281/zenodo.12205391.

Members of the Darwin Tree of Life Consortium are listed here: https://doi.org/10.5281/zenodo.4783558.
