# Supplementary material for: The genome sequence of the Long-tailed duck, Clangula hyemalis (Linnaeus, 1758)
Source: Wellcome Open Res. 2024 Oct 15;9:475. Originally published 2024 Aug 14. [Version 2] doi: 10.12688/wellcomeopenres.22779.2 (PMC11599803; doi:10.12688/wellcomeopenres.22779.2)
Supplement: Peer review history

# Reviewer report: Reviewer response for version 2

**DOI:** 10.21956/wellcomeopenres.25601.r111212

**Published:** 2024-11-26

**Authors:** Nagarjun Vijay

**Affiliations:** 1 Indian Institute of Science Education and Research Bhopal, Bhopal, Madhya Pradesh, India

**Summary**:

 The genome assembly of the Long-tailed duck generated as part of this effort is of high-quality with the use of PacBio HiFi and HiC sequencing data. Sufficient effort has been made to evaluate genome assembly quality and correct obvious errors. One could even call this a chromosome-level assembly as most of the scaffolds are assigned to chromosomes. 

 This genome is a good resource for the avian genomics community and the manuscript should be approved. However, the following comments are provided for further introspection.

**Comments about assembly QC**:

 In the "Genome sequence report" the authors state that "Manual assembly curation corrected 50 missing joins or mis-joins and two haplotypic duplications, reducing the scaffold number by 12.54%."

 The methods of manual assembly curation are not clear enough. What did this involve?? Looking at the scaffolds in IGV? Running some assembly validation tools? If so which tools? How is this step different from what is done using purge_dups? Do you mean the use of PretextView and HiGlass, How exactly was this done for this genome assembly? The link https://gitlab.com/wtsi-grit/rapid-curation provides a workflow, but the exact nature of corrections done for this assembly could not be found by this reviewer.

 The authors state "While not fully phased, the assembly deposited is of one haplotype.". How was this assessed? Did you run any haplotype verification tools?

**Minor comments**:

 1) It is mentioned that the genome will be annotated. What is the timeline for this? It is very curious that a genome is being assembled, but not annotated. While i understand this is the typical workflow of the project, annotation is seen as much a part of genome assembly as any other step.

 2) In my opinion, more rigorous assembly quality evaluation is justified. For instance, you should also use the following tools:

 (a) GAEP: a comprehensive genome assembly evaluating pipeline

 (b) Klumpy: A tool to evaluate the integrity of long-read genome assemblies and illusive sequence motifs

Are sufficient details of methods and materials provided to allow replication by others?

Yes

Is the rationale for creating the dataset(s) clearly described?

Yes

Are the datasets clearly presented in a useable and accessible format?

Yes

Are the protocols appropriate and is the work technically sound?

Yes

Reviewer Expertise:

genome assembly, speciation, population genetics

I confirm that I have read this submission and believe that I have an appropriate level of expertise to confirm that it is of an acceptable scientific standard.

# Reviewer report: Reviewer response for version 2

**DOI:** 10.21956/wellcomeopenres.25601.r110067

**Published:** 2024-11-26

**Authors:** Phred Benham

**Affiliations:** 1 University of Massachusetts Amherst Department of Biology (Ringgold ID: 117236), Amherst, Massachusetts, USA

The authors report a high-quality, chromosome level genome assembly for the Long-tailed Duck. The assembly was generated using sequencing approaches (PacBio and Hi-C) and assembly pipelines that are widely used by groups such as the vertebrate genomics project. I have only a few minor comments for the authors to address.

- The authors describe methods for generating RNAseq from muscle in the methods, but do not really mention these data elsewhere in the manuscript. Presumably this is for annotation (though only one tissue is not ideal). Can you state in methods what the RNA seq is for?
- Pg. 3 change show to shown.
- Pg. 6. You say samples were taken from a captive specimen. This makes it sound like it was a different bird. I think change the ‘a’ to ‘the’.
- Figure 3. Can you have a more descriptive y-axis label. Maybe instead of ERR…_cov, change to coverage or mean coverage.
- Pg. 7 delete ‘of’ before AMPure beads.

Are sufficient details of methods and materials provided to allow replication by others?

Yes

Is the rationale for creating the dataset(s) clearly described?

Yes

Are the datasets clearly presented in a useable and accessible format?

Yes

Are the protocols appropriate and is the work technically sound?

Yes

Reviewer Expertise:

Evolution, genomics, ornithology

I confirm that I have read this submission and believe that I have an appropriate level of expertise to confirm that it is of an acceptable scientific standard.

# Reviewer report: Reviewer response for version 2

**DOI:** 10.21956/wellcomeopenres.25601.r108706

**Published:** 2024-11-05

**Authors:** Jacqueline Smith

**Affiliations:** 1 The University of Edinburgh, Edinburgh, Scotland, UK

This paper describes the genome assembly of a (male) long-tailed duck ( *Clangula hyemalis*), representing 41 chromosomes (40 autosomes and the Z sex chromosome). Assembly was carried out using PacBio Hi-Fi data along with Hi-C technology. The scaffold N50 is 78Mb, with sequencing coverage around 43X.

 -I realize there is some debate on convention, but I would argue against capitalizing ‘Long-tailed duck’

 -Fig 1 – instead of showing the dead specimen that was sequenced, it would be more informative to show a typical picture of a male and female duck (especially considering the discussion of different plumage).

 -In the Background section, it would be interesting to add some comment about relative disease resistance of long-tailed ducks compared to other ducks.

 -Upon first mention of Pacific Biosciences, insert ‘(PacBio), as this abbreviation is subsequently used later in the paper.

 -Table 1 – there is no mention of RNAseq, so remove this information from the table.

 -Pg5 – BUSCO – in Fig 2 it mentions the aves_odb10 database, but in the text it says vertebrata_odb10. Which is correct?

 -I’m not sure how informative Figs 3 and 4 are. Maybe they could be combined into a smaller two-panel figure?

 -Methods – is there a reason the date is given in the format: 2022-01-06?

 -Methods – please provide company and location information for all mentioned kits and equipment.

 -Table 3 – are the numbers (1-40) purely based on size, or do they match chromosome numbers for previous duck assemblies?

 -It would be helpful to include another figure showing the alignment between the long-tailed duck and another duck (mallard?)

 -Pg9 – no need to mention ‘article in preparation’

 -Data availability – RNAseq is not presented in this paper and should not be mentioned here. It would be beneficial to have an annotation to supplement the genome as soon as possible. What RNAseq data is referenced? Data from the muscle sample that was collected? I would suggest that more tissues should be included for a more complete annotation, and that long-read transcriptomics be used if possible.

**Minor text corrections:**

 Pg3 – ‘egg lay’ as opposed to ‘egg laying’

 Pg3 – ‘at least 25,000 over four decades’

 Pg3 – ‘including plastics. In one study….’

 Pg3 – ‘declining population’ instead of ‘population declining’

 Pg5 – ‘while GC proportion and coverage across assembled scaffolds is shown in Figure 3.’

 Pg5 – ‘Most of the assembled sequence…’

Are sufficient details of methods and materials provided to allow replication by others?

Yes

Is the rationale for creating the dataset(s) clearly described?

Yes

Are the datasets clearly presented in a useable and accessible format?

Yes

Are the protocols appropriate and is the work technically sound?

Yes

Reviewer Expertise:

Avian genomics; disease resistance; environmental adaptation

I confirm that I have read this submission and believe that I have an appropriate level of expertise to confirm that it is of an acceptable scientific standard, however I have significant reservations, as outlined above.

# Reviewer report: Reviewer response for version 2

**DOI:** 10.21956/wellcomeopenres.25601.r108704

**Published:** 2024-11-04

**Authors:** Dinesh Velayutham

**Affiliations:** 1 Hamad Bin Khalifa University (HBKU), Doha, Qatar

The authors have done nice work to assemble chromosomal level assembly of *Clangula hyemalis* using hybrid approaches from long reads and Hi-C. The article has been described clearly in all sections. The data is also uploaded in NCBI.

**I have just couple of comments:**

 1. The authors could upload a gff3 file in NCBI (GCA_963989345.1), that will be beneficial to the research community than gdf file.

 2. If possible, any comparative genome analysis with Anas_platyrhynchos ((GCA_002743455.1) could be useful comparison for genus Clangula and Anas.

 I suggest to accept the article.

Are sufficient details of methods and materials provided to allow replication by others?

Yes

Is the rationale for creating the dataset(s) clearly described?

Yes

Are the datasets clearly presented in a useable and accessible format?

Yes

Are the protocols appropriate and is the work technically sound?

Yes

Reviewer Expertise:

Pharmacogenomics, Bioinformatics and Population genomics

I confirm that I have read this submission and believe that I have an appropriate level of expertise to confirm that it is of an acceptable scientific standard.
